# Long Noncoding RNAs Expression Patterns Associated with Chemo Response to Cisplatin Based Chemotherapy in Lung Squamous Cell Carcinoma Patients

**DOI:** 10.1371/journal.pone.0108133

**Published:** 2014-09-24

**Authors:** Zhibo Hou, Chunhua Xu, Haiyan Xie, Huae Xu, Ping Zhan, Like Yu, Xuefeng Fang

**Affiliations:** 1 First Department of Respiratory Medicine, Nanjing Chest Hospital, Medicine School of Southeast University, Nanjing, Jiangsu, China; 2 Clinical Center of Nanjing Respiratory Diseases and Imaging, Nanjing, Jiangsu, China; 3 Department of Pharmacy, The First Affiliated Hospital of Nanjing Medical University, Nanjing, Jiangsu, China; 4 Department of Medical Oncology, Second Affiliated Hospital, Zhejiang University College of Medicine, Hangzhou, Zhejiang, China; CSIR Institute of Genomics and Integrative Biology, India

## Abstract

**Background:**

There is large variability among lung squamous cell carcinoma patients in response to treatment with cisplatin based chemotherapy. LncRNA is potentially a new type of predictive marker that can identify subgroups of patients who benefit from chemotherapy and it will have great value for treatment guidance.

**Methods:**

Differentially expressed lncRNAs and mRNA were identified using microarray profiling of tumors with partial response (PR) vs. with progressive disease (PD) from advanced lung squamous cell carcinoma patients treated with cisplatin based chemotherapy and validated by quantitative real-time PCR (qPCR). Furthermore, the expression of AC006050.3-003 was assessed in another 60 tumor samples.

**Results:**

Compared with the PD samples, 953 lncRNAs were consistently upregulated and 749 lncRNAs were downregulated consistently among the differentially expressed lncRNAs in PR samples (Fold Change≥2.0-fold, *p* <0.05). Pathway analyses showed that some classical pathways, including “Nucleotide excision repair,” that participated in cisplatin chemo response were differentially expressed between PR and PD samples. Coding-non-coding gene co-expression network identified many lncRNAs, such as lncRNA AC006050.3-003, that potentially played a key role in chemo response. The expression of lncRNA AC006050.3-003 was significantly lower in PR samples compared to the PD samples in another 60 lung squamous cell carcinoma patients. Receiver operating characteristic curve analysis revealed that lncRNA AC006050.3-003 was a valuable biomarker for differentiating PR patients from PD patients with an area under the curve of 0.887 (95% confidence interval 0.779, 0.954).

**Conclusions:**

LncRNAs seem to be involved in cisplatin-based chemo response and may serve as biomarkers for treatment response and candidates for therapy targets in lung squamous cell carcinoma.

## Background

Lung cancer is a leading cause of cancer-related deaths worldwide, with non-small cell lung cancer (NSCLC) accounts for approximately 85% of all cases [1]. Most NSCLC patients are diagnosed at an advanced stage and have a 5-year survival rate of less than 20% [1]. Squamous cell carcinoma (SCC) is the second most common type of lung cancer, accounting for over 30% of NSCLC [2]. Encouraging new targeted agents have afforded benefits to lung adenocarcinoma (ADC) patients. Unfortunately, targeted agents developed for lung ADC are largely ineffective against lung SCC. Currently, the standard treatment for lung SCC remains a doublet of cisplatin plus one of the new agents other than pemetrexed [3]–[5]. However, there is large variability among individuals in response to treatment with cisplatin based chemotherapy [6], [7]. This highlights the importance of exploring new biomarkers that can predict cisplatin-based treatment efficacy for lung SCC.

Human genome is comprised of ∼1.2% protein coding genes and that ∼90% of the genome is transcribed as non-coding RNA (ncRNA)[8]. The ncRNAs can be divided into two major classes: small noncoding RNAs (<200 bp), such as microRNA, and long noncoding RNAs (lncRNAs;>200 bp) according to their transcript size. lncRNAs can be classified into exonic lncRNAs, intronic lncRNAs, intergenic lncRNAs (also known as large intergenic non-coding RNAs, lincRNAs) and overlapping lncRNAs in accordance with their location relative to the protein-coding transcripts[9]. LncRNAs have been implicated in carcinogenesis and cancer progression [10]–[15]. lncRNAs can act as tumor oncogenes or tumor suppressors just like protein coding genes or miRNA.

Recent studies suggest that lncRNAs also play a significant role in chemotherapy sensitivity and some lncRNAs has now been associated with chemotherapy sensitivity phenotypes in cancer. The lnRNA H19 gene could induce P-glycoprotein expression and MDR1-associated drug resistance in liver cancer cells through regulation of MDR1 promoter methylation [16]. LncRNAs are differently expressed between lung ADC A549 and A549/CDDP cells, many of which could regulate cisplatin resistance through different mechanisms. LnRNAAK126698 was found to confer cisplatin resistance by targeting the Wnt pathway [17]. LncRNA HOTAIR was observed to be significantly downregulated in cisplatin-responding lung ADC tissues and contributes to cisplatin resistance of lung ADC cells via regualtion of p21^WAF1/CIP1^ expression [18].

Owing to its possible effect on cisplatin resistance, we anticipated whether lncRNAs might influence tumor response to cisplatin based chemotherapy in lung SCC. The identification of lncRNAs that predict either sensitivity or resistance to cisplatin based chemotherapy is of great importance to individualized treatment of lung SCC.

In this study, we profiled lncRNA and mRNA expression in lung SCC patients having either partial response or progressive disease after cisplatin based chemotherapy. An integrative analysis combining lncRNA and mRNA changes within co-expression networks was performed to explore genes that may be related to cisplatin sensitivity in lung SCC. Several of different expressions of lncRNAs and mRNA were further validated by quantitative real-time PCR (qPCR) in lung SCC tissue samples. lncRNAs expression profiles may provide new molecular biomarkers for predicting responding to cisplatin based chemotherapy of lung SCC.

## Methods

### Patient Samples

All collected snap-frozen tissue samples used in this study were obtained by biopsy through bronchoscope or percutaneous lung biopsy under computerized tomography scan from primary sites of advanced stage lung SCC patients at Nanjing Chest Hospital and Second Affiliated Hospital of Zhejiang University College of Medicine during January 2009 and January 2013. All patients were histopathologically diagnosed by at least two independent senior pathologists. All of the tumors were unresectable and no patient underwent radiotherapy or chemotherapy prior to biopsy. Front-line chemotherapy comprised cisplatin 75 mg/m^2^ on days 1, and gemcitabine 1000 mg/m^2^ on days 1, 8, or docetaxel 75 mg/m2 on days 1 every 21 days for a maximum of 4 cycles. Response to therapy was defined by thoracic computerized tomography scan according to Response Evaluation Criteria In Solid Tumors (RECIST 1.1) [19]. Objective tumor response for target lesions are classed as: complete Response (CR), partial response (PR), progressive disease (PD), and stable disease (SD). In this study, PR was considered as sensitive and PD was considered as resistant. Tissue samples were obtained after patients' written informed consent under a general tissue collection protocol approved by The Research Ethics Committee of the Nanjing Chest Hospital and The Research Ethics Committee of Second Affiliated Hospital of Zhejiang University College of Medicine.

### LncRNA microarray and Computational Analysis

#### Samples

Total RNA was extracted with TRIzol reagent (Invitrogen, Carlsbad, CA, USA) according to the manufacturer's protocol. RNA quantity and quality were measured by NanoDrop ND-1000 spectrophotometer (PeqLab, Erlangen, Germany). Total RNA integrity was assessed by Agilent 2100 Bioanalyzer (Agilent Technologies, Santa Clara, USA).

#### RNA microarray

The Arraystar Human LncRNA Array v3.0 (Arraystar, Rockville, MD) was designed for profiling both lncRNAs and protein-coding RNAs in human genome. 33,045 lncRNAs were collected from the authoritative data sources including RefSeq, UCSC Knowngenes, Ensembl and many related literatures.

### RNA labeling and array hybridization

Sample labeling and array hybridization were performed according to the Agilent One-Color Microarray-Based Gene Expression Analysis protocol (Agilent Technologies, Santa Clara, USA) with minor modifications. Briefly, mRNA was purified from total RNA after removal of rRNA using mRNA-ONLY Eukaryotic mRNA Isolation Kit (Epicentre Biotechnologies, Madison, Wisconsin, USA). Then, each sample was amplified and transcribed into fluorescent cRNA along the entire length of the transcripts without 3′ bias utilizing a random priming method. The labeled cRNAs were purified by RNeasy Mini Kit (Qiagen, Inc., Valencia, CA). The concentration and specific activity of the labeled cRNAs (pmol Cy3/µg cRNA) were measured by NanoDrop ND-1000. 1 µg of each labeled cRNA was fragmented by adding 5 µl 10 × Blocking Agent and 1 µl of 25 × Fragmentation Buffer, then heated the mixture at 60°C for 30 min, finally 25 µl 2 × GE Hybridization buffer was added to dilute the labeled cRNA. 50 µl of hybridization solution was dispensed into the gasket slide and assembled to the LncRNA expression microarray slide. The slides were incubated for 17 hours at 65°C in an Agilent Hybridization Oven. The hybridized arrays were washed, fixed and scanned with using the Agilent DNA Microarray Scanner (part number G2505C).

#### Data analysis

Agilent Feature Extraction software (version 11.0.1.1) was used to analyze acquired array images. Quantile normalization and subsequent data processing were performed with using the GeneSpring GX v11.5.1 software package (Agilent Technologies, Santa Clara, USA). After quantile normalization of the raw data, lncRNAs and mRNAs that at least 10 out of 10 samples have flags in Present or Marginal ("All Targets Value") were chosen for further data analysis. Differentially expressed lncRNAs and mRNAs with statistical significance between the two groups were identified through Volcano Plot filtering. Log fold-change means log2 value of absolute fold-change. Fold-change and *p* value are calculated from the normalized expression. Hierarchical Clustering was performed using the Agilent GeneSpring GX software (version 11.5.1). The microarray data have been deposited in National Center for Biotechnology Information (NCBI) Gene Expression Omnibus (GEO) database and are accessible through GEO series accession number GSE59245 (http://www.ncbi.nlm.nih.gov/geo/query/acc.cgi?acc=GSE59245).

### qPCR

The expression of lncRNA or mRNA was detected by qPCR. The primers are listed as Table S1. β-actin was used as an internal control. The primers for β-actin were as follows: the forward primer 5′-AGCGAGCATCCCCCAAAGTT-3′ and the reverse primer 5′-GGGCACGAAGGCTCATCATT-3′. qPCR was performed using the SYBR Green (TaKaRa Bio Inc., Dalian, China) dye detection method on ABI PlusOne PCR instrument under default conditions: 95°C for 10 sec, and 40 cycles of 95°C for 5 s and 55°C for 31 S. Relative gene expression levels were analyzed by the 2^−ΔCt^ method, where ΔCt  =  Ct_target_− Ct_β-actin_ [20].

### Functional group analysis

Base on the latest KEGG (Kyoto Encyclopedia of Genes and Genomes) database (http://www.genome.jp/kegg/), we provide pathway analysis for differentially expressed mRNAs. This analysis allows us to determine the biological pathway that there is a significant enrichment of differentially expressed mRNAs. The *p*-value (EASE-score, Fisher-*P* value or Hypergeometric-*P* value) denotes the significance of the Pathway correlated to the conditions. The recommend *p*-value cut-off is 0.05.

### Construction of the Coding-non-coding Gene Co-expression Network

Gene co-expression network was constructed according to the specific expression lncRNAs and mRNAs [21]. The median gene expression value was used to represent the expression of the same coding gene with different transcripts. The primary lncRNA expression value was adopted with no particular processing. To normalize signal intensity of specific expression genes, we remove the subset of data that shown the differential expression of lncRNA and mRNA according to the primary lists from the microarray results. Pearson correlation coefficient (PCC) was calculated and the R value was used to compute the correlation coefficient of the PCC between lncRNAs and coding genes. LncRNAs and mRNAs with Pearson correlation coefficients not less than 0.99 were selected as significant correlation pairs to draw the co-expression network using Cytoscape. In the network, a regular hexagon node represents lncRNA, circular node represents the coding gene. A brown node represents an over-regulated lncRNA or mRNA and a blue node represents an under-regulated lncRNA or mRNA. The solid lines indicate a positive correlation and the dashed line indicates a negative correlation.

### Statistical Analysis

The GraphPad Prism 6.0 (GraphPad Software, LaJolla, CA) was used for statistical analysis. Data are expressed as the means ± SD. For a single comparison of 2 groups, Student's t test was used. Differences were considered statistically significant at *P*<0.05. A receiver operating characteristic (ROC) curve was performed by MedCalc software (version 11.4; Broekstraat, Mariakerke, Belgium).

## Results

### Differentially expressed lncRNAs

**Figure 1:**
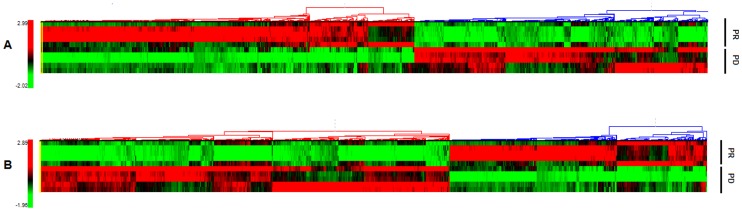
Heatmaps of lncRNA and mRNA expression patterns. RNA expression is depicted according to treatment response. (A) Hierarchical Clustering for “Differentially expressed lncRNAs”. (B) Hierarchical Clustering for “Differentially expressed mRNAs”. “Red” indicates high relative expression, and “green” indicates low relative expression.

We profiled the expression of lncRNAs in tumors from patients with advanced SCC subsequently treated with cisplatin based chemotherapy. Five patients experienced PR and five patients experienced PD according to the RECIST criteria. The basic information of the ten patients was shown in Table S2. The expression profiles of lncRNAs in two groups were shown by calculating log fold change PR/PD, positive value indicates upregulation and negative value indicates downregulation. Differentially expressed lncRNAs with statistical significance were identified by a Volcano Plot filtering between PR and PD groups (Fold Change cut-off: 2.0, *P*-value<0.05). Compared with the PD samples, 953 lncRNAs were consistently upregulated, and 749 lncRNAs were downregulated consistently among the differentially expressed lncRNAs in PR samples. Hierarchical clustering analysis was used to arrange samples into groups based on their expression levels (Figure 1A). The expression levels of the 20 top ranked lncRNAs in the different samples (PR vs. PD) are listed in Table 1.

**Table 1:** Deregulated lncRNAs detected using microarray in 5 PR and 5 PD lung SCC patients.

Down-regulated in PR group					Up-regulated in PR group				
Genbank accession	Gene Symbol	*P*-value	Fold change*	FDR	Genbank accession	GeneSymbol	*P*-value	Fold change*	FDR
ENST00000583748	AC022596.6	1.19E-05	122.41	0.0012	NR_026892	AFAP1-AS1	0.0001	142.91	0.0047
ENST00000425692	AC022596.6	4.85E-06	58.58	0.0007	ENST00000566942	RP11-284N8.3	0.0001	45.79	0.0047
ENST00000584612	KRT16P2	0.0043	56.25	0.0384	ENST00000562112	NAPSB	0.0062	32.73	0.0458
ENST00000581210	KRT16P1	0.0014	48.55	0.0200	ENST00000567369	CTA-363E6.2	5.29E-07	19.47	0.0002
ENST00000579062	KRT16P2	0.0042	48.47	0.0381	ENST00000579713	RP11-403A21.2	2.95E-07	15.47	0.0002
ENST00000395675	FOXO3B	0.0002	39.05	0.0056	ENST00000565118	ABCC6P1	0.0002	14.60	0.0069
ENST00000446115	RP11-439L18.3	0.0006	36.97	0.0121	ENST00000450920	SRGAP3-AS2	0.0040	13.80	0.0368
NR_024538	GSTM4	0.0003	29.87	0.0072	TCONS_00003617	XLOC_001406	0.0075	13.72	0.0509
ENST00000458343	KRT42P	0.0015	29.45	0.0207	ENST00000433329	HBBP1	0.0006	12.58	0.0125
ENST00000555300	RP11-104E19.1	7.8E-07	28.86	0.0003	uc010jub.1	AK293020	0.0001	11.98	0.0044
ENST00000583364	AC015818.3	1.91E-07	28.54	0.0002	ENST00000562490	RP11-102F4.3	2.32E-07	10.89	0.0002
ENST00000578693	AC006050.3	0.0007	24.61	0.0130	ENST00000564038	RP11-1223D19.1	0.0002	10.67	0.0066
ENST00000412143	PSORS1C3	0.0003	24.57	0.0085	hsa-mir-940	MI0005762	0.0006	9.71	0.0115
ENST00000326333	KRT17P2	0.0006	24.09	0.0119	ENST00000466677	RP4-555L14.5	0.0028	9.27	0.0293
ENST00000579363	AC022596.2	0.0014	23.44	0.0195	NR_024344	LOC283174	0.0022	8.40	0.0261
ENST00000420566	AC006050.3	0.0020	23.12	0.0243	NR_038386	LOC728537	0.0001	7.59	0.0042
ENST00000554221	LINC00520	0.0018	22.79	0.0231	uc022cjh.1	IGL@	0.0019	7.11	0.0235
ENST00000560054	RP11-499F3.2	0.0056	22.71	0.0439	uc001lku.1	AK125699	3.12E-06	7.09	0.0006
ENST00000577817	AC006050.3	0.0011	21.01	0.0173	NR_038200	M1	0.0056	6.94	0.0440
ENST00000584833	AL353997.3	0.0016	20.29	0.0211	ENST00000570700	RP11-473M20.11	0.0034	6.86	0.0335

*Log2 (PR/PD).

### LncRNA Classification and Subgroup Analysis

#### Enhancer lncRNAs profiling

LncRNAs with enhancer-like function are identified using GENCODE annotation of the human genes [22], [23]. The consideration of selection of lncRNAs with enhancer-like function exclude transcripts mapping to the exons and introns of annotated protein coding genes, the natural antisense transcripts, overlapping the protein coding genes and all known transcripts. Fifty-one differentially expressed enhancer-like lncRNAs and their nearby coding genes (distance, 300 kb) were showed in Table S3.

#### HOX cluster profiling

In current study, the profiling data of all probes targeting 407 discrete transcribed regions in the four human HOX loci for both lncRNAs and coding transcripts was presented in the Table S4 [24]. These data suggested that 30 coding transcripts could be detected in SCC tissues with 17 of them differentially expressed. Then, about 34 lncRNAs transcribed were detected in SCC tissues and 18 of them were found differently expressed in human HOX loci.

#### LincRNAs profiling

lincRNAs are a subtype of lncRNAs, which are transcribed from intergenic regions [2]. Previous studies found that lincRNAs could regulate the expression of neighbouring genes and distant genomic sequences, thus play key roles in certain biological processes [25], [26]. All probes for lincRNAs in microarray were calculated by genomic coordinates [13], [27]. 405 differentially expressed lincRNAs and nearby coding gene pairs (distance <300 kb) between PR and PD groups were showed in Table S5.

### Differentially expressed mRNAs

From the mRNA expression profiling data, a total of 16,851 mRNAs were identified in the samples through microarray analysis. Compared with PD group, 1223 mRNAs were consistently upregulated, and 1947 mRNAs were consistently downregulated in PR group. The expression levels of the 20 top ranked mRNAs in the different samples (PR vs. PD) are listed in Table 2. The Hierarchical clustering analysis indicated the relationships among the mRNA expression patterns that were present in the samples (Figure 1B).

**Table 2:** Deregulated mRNAs detected using microarray in 5 PR and 5 PD lung SCC patients.

Down-regulated in PR group					Up-regulated in PR group				
Genbank accession	Gene Symbol	*P*-value	Fold change*	FDR	Genbank accession	GeneSymbol	*P*-value	Fold change*	FDR
NM_207392	KRTDAP	5.97E-08	210.97	5.92E-05	ENST00000304749	CST1	0.0038	31.22	0.0244
NM_003125	SPRR1B	9.72E-05	138.34	0.0027	NM_001145077	LRRC10B	0.012	23.30	0.0480
ENST00000368750	SPRR2E	0.0045	127.54	0.0273	NM_001904	CTNNB1	2.11E-05	20.46	0.0012
ENST00000360379	SPRR2D	3.50E-05	104.87	0.0015	NM_001008219	AMY1C	0.0041	19.80	0.0255
NM_005988	SPRR2A	0.0051	93.73	0.0290	NM_178452	DNAAF1	0.011	19.07	0.0453
NM_020299	AKR1B10	0.0017	90.00	0.0149	NM_025244	TSGA10	0.0038	17.99	0.0246
ENST00000368789	LCE3E	3.93E-05	86.40	0.0016	NM_000699	AMY2A	0.0065	17.67	0.0335
NM_001017418	SPRR2B	0.00098	85.02	0.0109	ENST00000305904	DYNLRB2	0.0099	17.31	0.0429
NM_001080538	AKR1B15	0.0230	84.25	0.0230	NM_001306	CLDN3	0.0072	16.82	0.0354
NM_032330	CAPNS2	0.0066	69.87	0.0338	NM_001008218	AMY1B	0.0065	15.94	0.0335
NM_182502	TMPRSS11B	4.28E-06	66.42	0.0005	NM_147169	C9orf24	0.012	15.79	0.0473
NM_001014450	SPRR2F	0.0016	64.79	0.0148	ENST00000379133	C9orf24	0.011	15.57	0.0447
ENST00000171111	KEAP1	0.0027	59.87	0.0199	NM_024687	ZBBX	0.0078	15.52	0.0371
NM_002638	PI3	0.0015	56.93	0.0141	NM_152784	CATSPERD	0.0013	15.40	0.0128
NM_012397	SERPINB13	0.00059	50.69	0.0080	NM_173554	C10orf107	0.0075	15.16	0.0365
NM_005218	DEFB1	5.29E-07	47.29	0.0001	NM_015668	RGS22	0.0064	14.08	0.0332
ENST00000368733	S100A8	5.95E-05	46.06	0.0021	NM_001935	DPP4	0.0020	13.52	0.0168
NM_000526	KRT14	2.91E-07	42.90	0.0001	NM_001098517	CADM1	0.0004	13.04	0.0062
NM_004988	MAGEA1	7.86E-07	38.92	0.0002	NM_000900	MGP	0.0002	10.71	0.0041
NM_002639	SERPINB5	0.0082	37.14	0.0385	ENST00000288710	CCDC164	0.0111	10.47	0.046

*Log2 (PR/PD).

### Validation of the microarray data using qPCR

**Figure 2:**
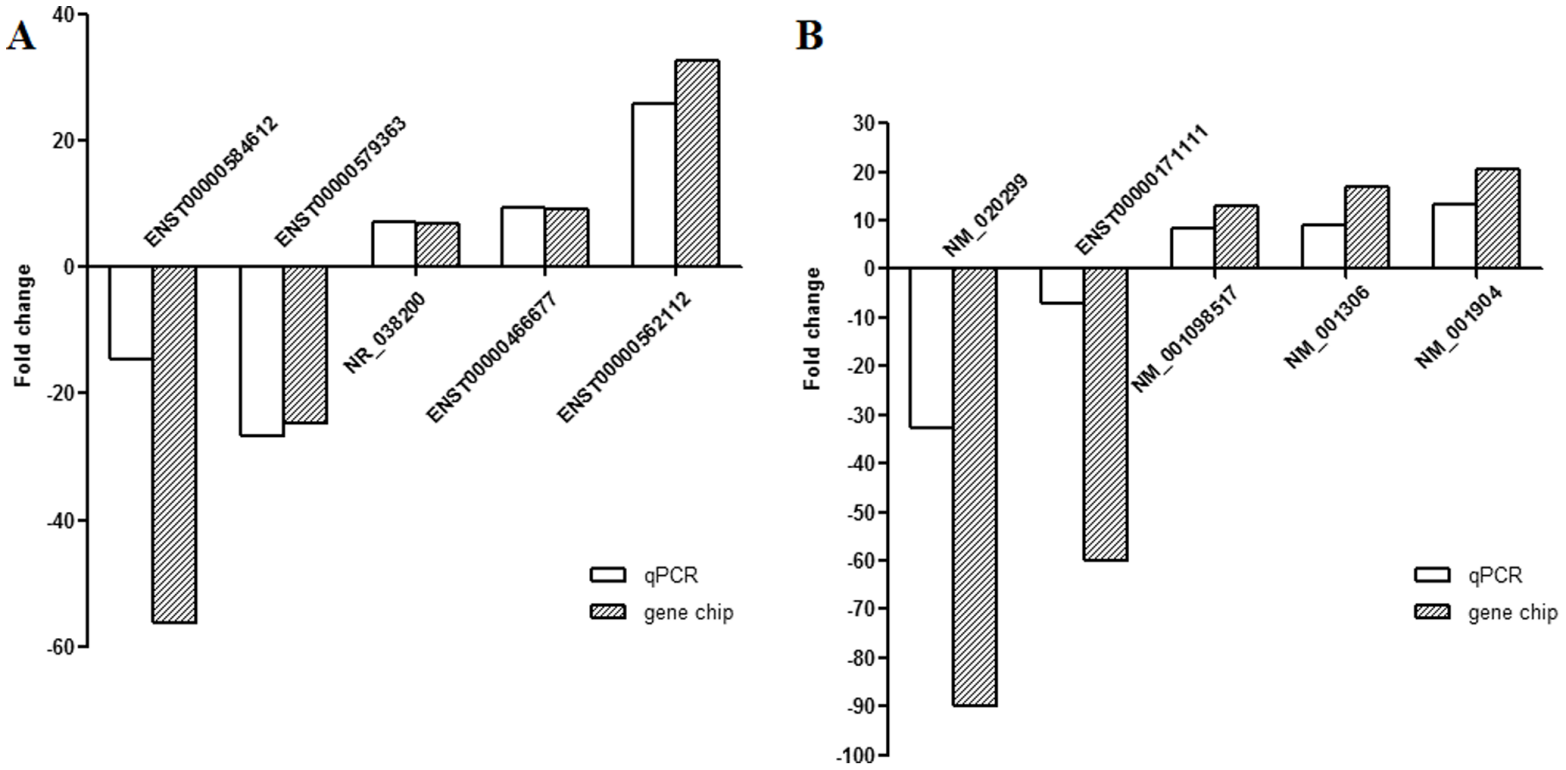
Validation of microarray data by qPCR. The differential expression of 5 lncRNAs (A) or 5 mRNAs (B) in samples of 10 patients by microarray was validated by qPCR. The relative expression level in PR samples was normalized by the PD samples. The heights of the columns in the chart represent the median fold changes (PR/PD) in expression across the patients for each of the validated lncRNAs or mRNAs. Fold changes were calculated by the 2^−ΔCt^ method. Fold changes  = mean2^−ΔCt^ (PR)/mean2^−ΔCt^ (PD), where ΔCt  =  Ct_target_−Ct_β-actin_.

Five lncRNAs (ENST00000584612, ENST00000579363, NR_038200, ENST00000466677 and ENST00000562112) and five mRNAs (NM_020299, ENST00000171111, NM_001098517, NM_001306 and NM_001904) were randomly selected to validate the microarray consistency by using qPCR. The results demonstrated that lncRNA ENST00000584612 and ENST00000579363 were downregulated and that NR_038200, ENST00000466677 and ENST00000562112 were upregulated in the PR samples compared with PD samples (Figure 2A). For mRNA, NM_020299 and ENST00000171111 were downregulated and that NM_001098517, NM_001306 and NM_001904 were upregulated in the PR samples compared with PD samples (Figure 2B). These above qPCR results are consistent with microarray data.

### Pathway analysis

**Figure 3:**
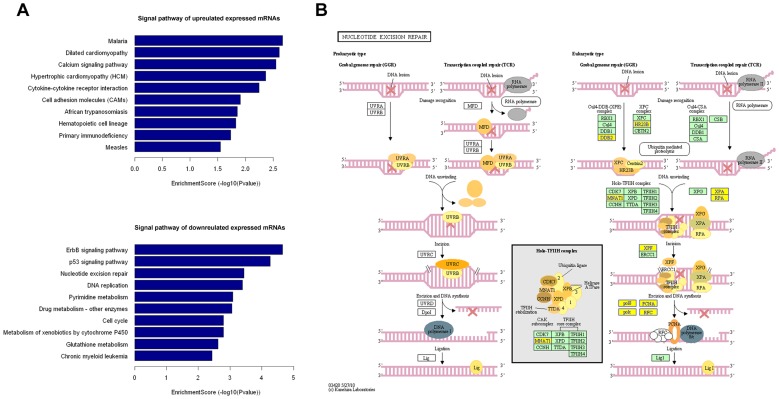
Pathway analysis of the differentially expressed mRNAs. (A) Signaling pathways of upregulated mRNAs and downregulated mRNAs. The bar plot shows the top ten Enrichment score (−log10 (*P*-value)) value of the significant enrichment pathway. (B) The "Nucleotide excision repair" signal pathway shows modulation in repair of nonspecific DNA damage and is associated with cisplatin sensitivity. Yellow marked nodes are associated with downregulated genes, orange marked nodes are associated with upregulated or only whole dataset genes, and green nodes have no significance.

Pathway analysis indicated that 32 pathways corresponded to underregulated transcripts in PR group. Among the 32 pathways, we found that several enriched networks including "Nucleotide excision repair", "Glutathione metabolism", "Drug metabolism-cytochrome P450", "Metabolism of xenobiotics by cytochrome P450", "Drug metabolism-other enzymes" and "Calcium signaling pathway" were associated with chemotherapeutic drugs metabolism (Figure 3A). Of note, "Nucleotide excision repair" has been reported intensively to be correlated with cisplatin sensitivity (Figure 3B) [28], [29]. Furthermore, Pathway analysis showed that 15 pathways corresponded to upregulated transcripts in PD group. One of these pathways, the gene category "Calcium signaling pathway", has been reported to be involved in the cisplatin resistance [30], [31] (Figure 3A).

### Co-expression network

**Figure 4:**
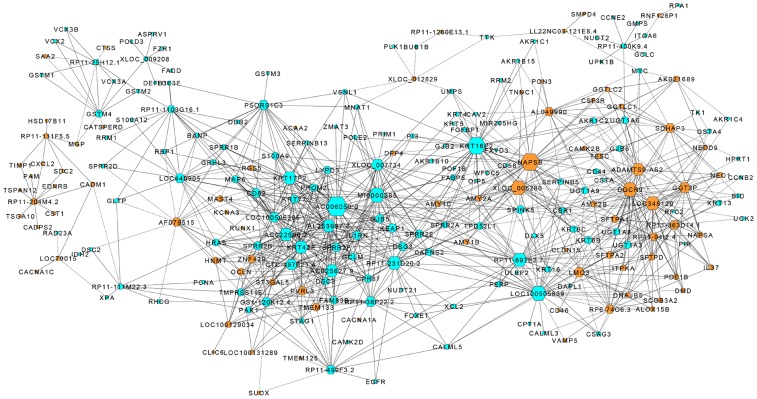
LncRNA-mRNA-network was constructed based on the correlation analysis between the differential expressed lncRNAs and mRNAs. In the network, a regular hexagon node represents lncRNA, circular node represents the mRNA. A brown node represents an upregulated lncRNA or mRNA and a blue node represents a downregulated lncRNA or mRNA.

Coding-non-coding gene co-expression network analysis was undertaken to explore which gene played a critical role in cisplatin resistance. The more important role the gene played, the more central the gene is within the network. Co-expression network was constructed to cluster lncRNAs and coding mRNAs into phenotypically relevant modules based on the correlation analysis between the differential expressed lncRNAs and mRNAs (Figure 4). Among this co-expression network, 49 lncRNAs and 186 mRNAs composed the CNC network node. Two hundred and thirty-five network nodes made associated 1063 network pairs (700 positive correlations and 363 negative correlations) of co-expression lncRNAs and mRNAs. The results indicated that many lncRNAs such as AC006050.3, NAPSB, KRT16P2, XLOC_005280 and MI0000285 potentially play important role in the network. AC006050.3 has 3 transcripts, such as AC006050.3-001, AC006050.3-002, and AC006050.3-003. All the 3 transcripts are downregulated in PR group compared to PD group (Table 1). With co-expression network, lncRNA AC006050.3-003 (ENST00000578693) expression level correlated with many members of the NER pathway such as DDB2, POLE2 and MNAT1 (Table S6). LncRNA AC006050.3-003 might play key role in cisplatin chemo response and was selected for further study.

### Potential predictor values of AC006050.3-003

**Figure 5:**
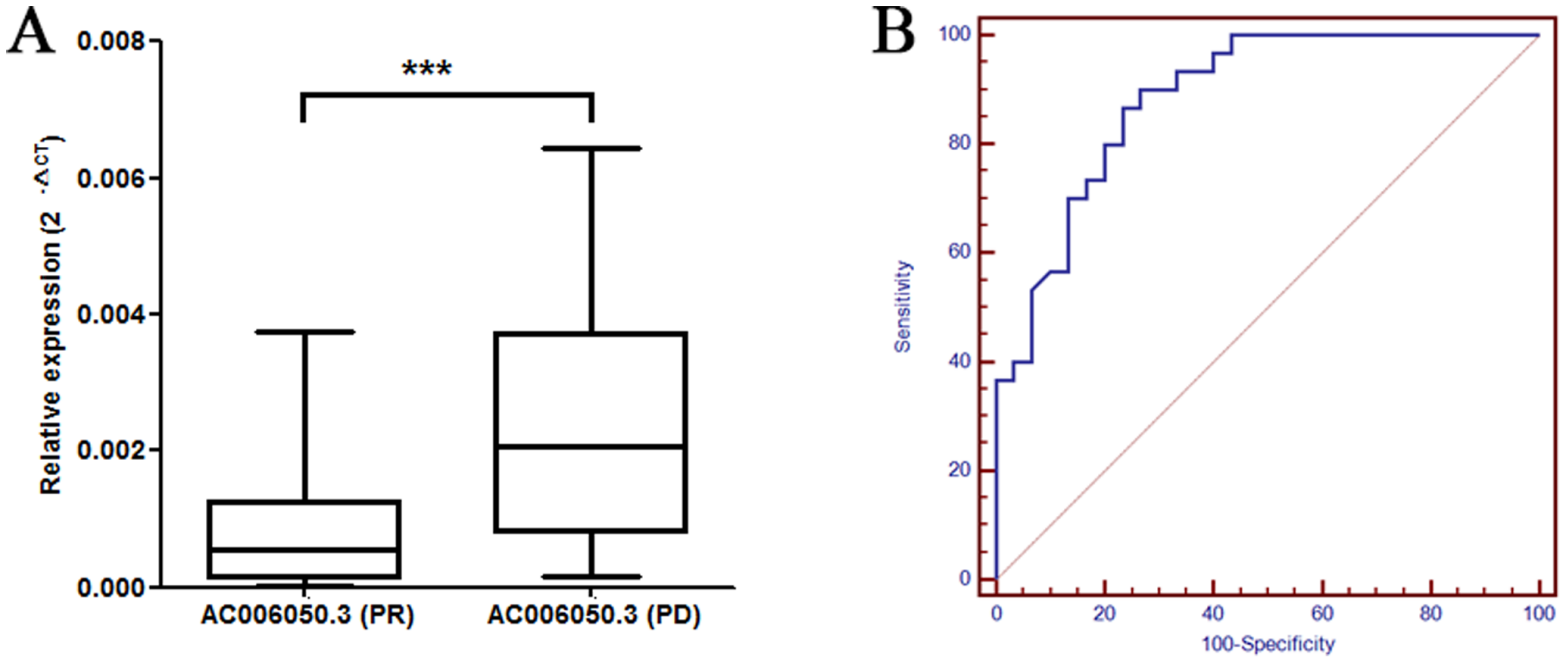
The expression of lncRNA AC006050.3-003 was validated by qPCR in samples of 60 patients with lung SCC stratified according to the chemo response (PR vs. PD) following cisplatin based chemotherapy. (A) lncRNA AC006050.3-003 was aberrantly expressed between PC and PD patients. The term 2^-ΔCt^ was used to describe the relative expression level of lncRNA (ΔCt  =  Ct_target_−Ct_ β-actin_). ***P<0.001 for patients with PD versus patients with PR (Student's t-test). (B) ROC analysis of the ability of lncRNA AC006050.3-003 levels to discriminate between PR and PD patients with lung SCC receiving cisplatin based chemotherapy.

The expression of lncRNA AC006050.3-003 was next detected by qPCR in the other 60 lung SCC patients received cisplatin based chemotherapy (Figure 5A). The expression of lncRNA AC006050.3-003 was significantly lower in PR samples compared with the PD samples. These results indicated that lncRNA AC006050.3-003 was aberrantly expressed between PC and PD patients. We next performed an analysis to identify whether lncRNA AC006050.3-003 expression could predict the effect of cisplatin chemo response. As shown in Figure 5B, ROC curve analysis revealed that lncRNA AC006050.3-003 was a valuable biomarker for differentiating PR patients from PD patients with an area under the curve (AUC) of 0.887 (95% confidence interval 0.779, 0.954). These results suggested that lower expression of lncRNA AC006050.3-003 might correlate with sensitivity to chemotherapy in lung SCC patients.

## Discussion

In the present study, we performed lncRNA expression profiling of tumor samples from patients with advanced lung SCC that showed PR or PD, following cisplatin based chemotherapy. The lncRNA profiling identified a set of differentially expressed lncRNAs that were correlated with chemotherapy response. There are 953 upregulated lncRNAs and 749 downregulated lncRNAs that were significantly differentially expressed (≥2.0-fold) in PR group compared to PD group.

The qPCR results validate that expression of lncRNAs are consistent with the data of microarray. There was distinctive expression of lncRNAs between PR and PD samples. It was likely to provide potential way to distinguish PR group from PD group and provide new biomarkers to predict cisplatin sensitivity for lung SCC individualized treatment. The differentially expressed lncRNAs and nearby coding gene pairs identified here may provide novel path for better understanding of the molecular basis of cisplatin resistance in lung SCC.

The lncRNA expression microarray used in this study included five subgroups: Enhancer lncRNAs, Rinn lincRNAs, HOX cluster, lincRNAs nearby coding gene, and Enhancer lncRNAs nearby coding gene. HOX lncRNAs and the lncRNAs with enhancer-like function are two special subgroups of lncRNAs. Previous studies have shown that there is deregulation of HOX gene expression in various of cancers including lung cancer [32], [33]. HOX lncRNAs are intergenic and are transcribed in the direction opposite to the HOX genes [34], [35]. Many studies have reported that HOX lncRNAs are implicated in transcriptional regulation of neighboring *HOX* genes and found to involve in the occurrence and development of cancers [10], [14], [36], [37]. For example, HOTAIR (Hox transcript antisense intergenic RNA) is significantly upregulated in NSCLC tissues, and involves in NSCLC cell proliferation and metastasis, partially via the downregulation of HOXA5 [12], [38]. In current study, we showed that both lncRNAs and coding transcripts transcribed of HOX loci were differentially expressed between PC and PD samples. These data suggested that differentially expressed coding transcripts and lncRNAs transcribed of HOX loci were correlated with cisplatin chemotherapy response in lung SCC. Our microarray also displayed a portion of differentially expressed enhancer like lncRNAs. LncRNAs with an enhancer-like function were identified in various human cell lines and were involved in cellular differentiation. Depletion of a number of enhancer lncRNAs could lead to decreased expression of their neighboring protein-coding genes, such as TAL1, Snai1 and Snai2 [23]. To uncover the precise mechanism by which enhancer lncRNAs function to enhance gene expression has potential to overcome cisplatin resistance.

Simultaneously, we also identified 1,224 differentially expressed protein coding mRNAs from the same gene chip. Pathway analysis was applied to explore which particular pathways were enriched in genes controlling the distinctive features of PR group compared to PD group based on the differentially expressed mRNAs from the microarray. KEGG annotation showed that 32 pathways corresponded to downregulated transcripts and 15 pathways corresponded to upregulated transcripts. Among the pathways that corresponded to downregulated transcripts in PR group compared to PD group, "Nucleotide excision repair" and "Glutathione metabolism" pathways were previously reported correlated with cisplatin sensitivity [28], [29]. Nucleotide excision repair (NER) systems are multistep enzymatic complexes involved in the repair of nonspecific DNA damage, such as cross linking, and chemical intra-/inter strand adduct formation. Cisplatin-DNA adducts is repaired primarily by the NER system [29]. Elevated glutathione may cause cisplatin resistance through inactivating cisplatin, increasing DNA repair, and decreasing cisplatin-induced oxidative stress [29]. In additional, "Drug metabolism-cytochrome P450", "Metabolism of xenobiotics by cytochrome P450", and "Drug metabolism-other enzymes" pathways are involved in drug metabolizing, affecting biotransformation of drugs. These drug metabolizing related pathways may contribute to interindividual variability in cisplatin response.

Gene co-expression networks were constructed to explore gene interactions [39], [40]. Firstly, based on the different expression levels of lncRNAs and mRNAs, we calculated the Pearson correlation coefficients. Next, we chose significant correlation pairs (PCC≥0.99) to construct a co-expression network to predict the possible relationship of lncRNAs and mRNAs. The degree of gene centrality, the number of links from one gene to another, determines its relative importance within the network analysis [41]. In this study, lncRNA AC006050.3, MI0000285, KRT16P12, NAPSB, XLOC_005280 fit to these characteristics. Yang et al. profiled the differently expressed lncRNAs and mRNAs between lung ADC A549 and A549/CDDP cells. Gene co-expression network identified that lncRNAs including BX648420, ENST00000366408, and AK126698 potentially played a key role in cisplatin resistance. But we did not see the different expression of these lncRNAs in our data. NSCLC can be further divided into 3 major histological subtypes: ADC, SCC and large cell carcinoma [1]. Despite sharing many biological features, SCC and AC subtypes differ in their cell of origin, location within the lung, and growth pattern, suggesting they are distinct diseases that develop cisplatin risistance through differential molecular mechanisms.

The centrality of lncRNA AC006050.3 indicates its relative importance in the lncRNA-mRNA co-expression network. With co-expression network, we found AC006050.3-003 expression level correlated with many members of the NER pathway. So we think that lncRNA AC006050.3-003 may play important role in cisplatin chemo response in lung SCC. Its gene is located in chromosome 17: 28,894,720–28,895,457. Its transcript length is 549 nt and has 3 exsons. Whether this lncRNA is associated with cancer are unknown yet (http://asia.ensembl.org/). Based on the co-expression network analysis, lncRNA AC006050.3-003 was selected to further evaluate the predictor value in determining clinical response of lung SCC patients receiving cisplatin based chemotherapy by qPCR through additional 60 lung SCC samples. The relative level of lncRNA AC006050.3-003 was significantly reduced in the PR group compared with the PD group. The expression of lncRNA AC006050.3-003 was potential marker to distinguish the sensitivity or resistance to cisplatin based chemotherapy in patients with lung SCC.

## Conclusion

Our study showed that numerous lncRNAs and mRNAs were differently expressed between PR and PD samples in advanced lung SCC patients following cisplatin based chemotherapy. LncRNAs seem to be involved in cisplatin based chemo response and may serve as biomarkers for chemo response and candidates for therapy targets in lung SCC. However, the sample size in this study is relative small and it requires a larger data set for further confirmation. The network of lncRNA-conding RNA interactions is very complex and thus requires further study to reveal the accurately molecular mechanisms by which lncRNAs function in cisplatin chemo response.

## Supporting Information

Table S1 **Primers for qPCR.**
(DOCX)

Table S2 **Basic medical records of ten patients.**
(DOCX)

Table S3 **Enhancer-like lncRNAs and their nearby coding genes.**
(XLS)

Table S4 **HOX cluster profiling.**
(XLS)

Table S5 **LincRNAs and their nearby coding gene data.**
(XLS)

Table S6 **Pearson correlation coefficients of AC006050.3-003.**
(XLSX)
