# Supplementary material for: Long Noncoding RNAs Expression Patterns Associated with Chemo Response to Cisplatin Based Chemotherapy in Lung Squamous Cell Carcinoma Patients
Source: PLoS One. 2014 Sep 24;9(9):e108133. doi: 10.1371/journal.pone.0108133 (PMC4176963; doi:10.1371/journal.pone.0108133)
Supplement: Table S1 — Primers for qPCR. (DOCX) [file pone.0108133.s001.docx]

Table S1 Primers of 11 genes

| Genbank accession | Gene name | Primer | 5’-3’ |
| --- | --- | --- | --- |
| ENST00000584612 | KRT16P2 | forward | ACGGGACTGGGAGTGATG |
|  |  | reverse | CGATGGTCTTGAAGTAGGGA |
| ENST00000579363 | AC022596.2 | forward | CACCTGGCTGCTGATGAC |
|  |  | reverse | AAGGATGGGAACTGGACT |
| NR_038200 | M1 | forward | TCATTCCCTTGACTTTGG |
|  |  | reverse | GGTAGGTAACACGGCTTC |
| ENST00000466677 | RP4-555L14.5 | forward | CACCGCTGACCTTACCTAC |
|  |  | reverse | TCTACAGTCCAGACTCAACCC |
| ENST00000562112 | NAPSB | forward | ATCCCTCTTCGTCAAGTCCA |
|  |  | reverse | ACCCGCCCAGTTCCATAC |
| NM_020299 | AKR1B10 | forward | TTGAGTGTCACCCATACC |
|  |  | reverse | ACTTCAGCGAAGAAAGAG |
| ENST00000171111 | KEAP1 | forward | ACTGTACCTGTTGAGGCACTTT |
|  |  | reverse | GCACATGATTCCCGCTTT |
| NM_001098517 | CADM1 | forward | ATTTCAGGGACTTCAGGC |
|  |  | reverse | ATCAGATTACGTGGTGGG |
| NM_001306 | CLDN3 | forward | CCTTCATCGGCAGCAACATC |
|  |  | reverse | CAGCAGCGAGTCGTACACCTT |
| NM_001904 | CTNNB1 | forward | ATTCTTGGCTATTACGACA |
|  |  | reverse | CCTCTATACCACCCACTT |
| ENST00000578693 | AC006050.3 | forward | TGGCTACCAACAGCGAACT |
|  |  | reverse | CCTCCAGGGATGCTTTCA |
