# Supplementary material for: Long Noncoding RNAs Expression Patterns Associated with Chemo Response to Cisplatin Based Chemotherapy in Lung Squamous Cell Carcinoma Patients
Source: PLoS One. 2014 Sep 24;9(9):e108133. doi: 10.1371/journal.pone.0108133 (PMC4176963; doi:10.1371/journal.pone.0108133)
Supplement: Table S2 — Basic medical records of ten patients. (DOCX) [file pone.0108133.s002.docx]

| Patient Number | age | sex | chemotherapy | clinical stage | RECIST Category |
| --- | --- | --- | --- | --- | --- |
| 141 | 60 | female | GP | Ⅲb | PR |
| 80 | 53 | male | GP | Ⅳ | PR |
| 164 | 58 | female | GP | Ⅳ | PR |
| 175 | 66 | female | GP | Ⅲb | PR |
| 177 | 58 | male | GP | Ⅲb | PR |
| 165 | 51 | male | GP | Ⅳ | PD |
| 169 | 70 | female | GP | Ⅳ | PD |
| 198 | 77 | female | GP | Ⅲb | PD |
| 69 | 72 | female | GP | Ⅲb | PD |
| 83 | 62 | male | GP | Ⅲb | PD |

Table S2 Basic medical records of ten patients
